# Life history profiles for 27 strepsirrhine primate taxa generated using captive data from the Duke Lemur Center

**DOI:** 10.1038/sdata.2014.19

**Published:** 2014-07-22

**Authors:** Sarah M Zehr, Richard G Roach, David Haring, Julie Taylor, Freda H Cameron, Anne D Yoder

**Affiliations:** 1 The Duke Lemur Center, Duke University, Durham, NC 27705, USA; 2 NESCent, Duke University, Durham, NC 27705, USA; 3 Department of Biology, Duke University, Durham, NC 27705, USA

## Abstract

Since its establishment in 1966, the Duke Lemur Center (DLC) has accumulated detailed records for nearly 4,200 individuals from over 40 strepsirrhine primate taxa—the lemurs, lorises, and galagos. Here we present verified data for 3,627 individuals of 27 taxa in the form of a life history table containing summarized species values for variables relating to ancestry, reproduction, longevity, and body mass, as well as the two raw data files containing direct and calculated variables from which this summary table is built. Large sample sizes, longitudinal data that in many cases span an animal’s entire life, exact dates of events, and large numbers of individuals from closely related yet biologically diverse primate taxa make these datasets unique. This single source for verified raw data and systematically compiled species values, particularly in combination with the availability of associated biological samples and the current live colony for research, will support future studies from an enormous spectrum of disciplines.

## Background and Summary

The Duke Lemur Center (DLC) here provides public availability of a life-history resource for the world’s largest collection of endangered primates. Since its establishment in 1966, the DLC has been dedicated to the study and conservation of prosimian primates—the lemurs, lorises, galagos, and tarsiers—with a special interest in the lemurs of Madagascar. The lemurs, lorises, and galagos together form the strepsirrhines, the sister clade to all other living primates (i.e., haplorhines). This report contains verified data for 3,627 individuals of the 27 strepsirrhine taxa shown in Table 1. The phylogeny, divergence times, and historical biogeography of this group are increasingly well-resolved^1,2^, giving biologists a secure historical framework within which to ask detailed questions relating to virtually all aspects of primate genotype and phenotype. The entire DLC historic collection is represented by nearly 4,200 individuals from over 40 taxa, and is the product of nearly 50 years of captive breeding, institutional exchange, and previously wild-caught animals obtained in collaboration with local authorities in their countries of origin. The current colony size is maintained at roughly 250 animals with 11 actively breeding species, and is the largest diverse collection of captive strepsirrhines worldwide. The endangered status of the species in the collection^3^, combined with regulations supporting their protection in the wild, make it extremely unlikely that a colony of this magnitude and diversity could ever be re-created.

**Figure 1:**
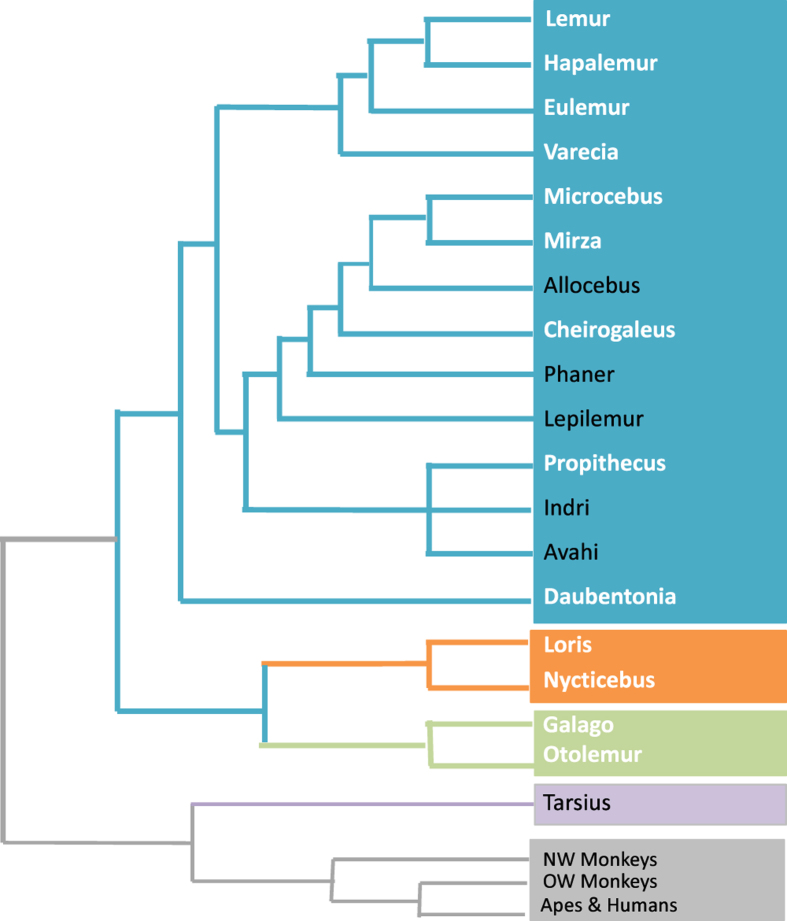
Primate phylogeny highlighting strepsirrhine genera included in this study. The recognized strepsirrhine genera and their phylogenetic relationships are shown^1,2,5,6^. Genera for which data are presented are shown in white, and include nine of 14 lemur genera, along with two genera from each of the loris and galago families.

**Figure 2:**
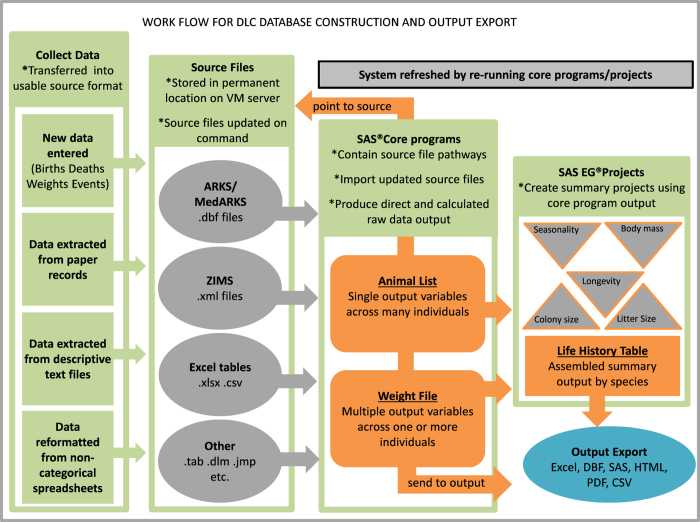
Workflow for DLC Database Construction. Output files contained in this database release are shown in orange. We can automatically refresh the output to include any newly entered data simply by re-running the core programs and secondary projects. By saving copies of the source files from any given date of update, older versions can be re-created by placing them in the source folder to which the core programs point. Data files presented in this paper are available in.csv format, but other possible output formats are indicated in the ‘Output Export’ cell.

Details of birth, death, reproduction, and growth, along with extensive husbandry and medical records, have been documented for each animal throughout the colony history. Taxa included in this report represent four of the five extant lemur families, and nine of 14 recognized lemur genera^4,5^, yielding good coverage of the Lemuroidae; representatives from the galago and loris lineages provide outgroups for the study of lemurs, and round out coverage of the strepsirrhine clade as shown in Figure 1. It is a remarkable collection of animal-associated data for a phylogenetically and biologically diverse assemblage of endangered primates. Until now, however, use of this information was both challenging and time-consuming because the data were not available in a uniform, easily searchable format—a problem that existed because data have been collected and stored in ways that span the range of technologies available at different points throughout the Center’s long history. We have extracted information from various incompatible formats, transferred data to usable source files, compiled them using SAS® software tools, and are now able to provide large amounts of colony data in flexible and analyzable formats. The workflow used to generate the output presented here is depicted in Figure 2.

We have generated a life history table that provides species data and summary statistics for variables relating to adult, young-adult, and neonatal body mass, birth and breeding season, litter size, age at reproduction, longevity, infant mortality, activity pattern, and numbers of live individuals who are available for study in our current colony or for which biological samples have been banked. The life history table was generated with values from the two accompanying data files: the DLC Animal List, containing single-copy variables for each of the 3,627 individuals included in this paper, and the DLC Weight File, containing 65,692 weight measurements from 2,174 of those individuals over time.

The data, used independently or when combined with the research accessibility of the DLC’s current live colony and the availability of affiliated biological samples for 1,012 of the individuals included in this paper, will support a large number of research projects across a diverse span of biological disciplines. As additional data from the historic colony are verified and data for newly arrived individuals obtained, future updates containing increasing numbers of variables, individuals and taxa will be made available.

## Methods

### Data source

The Duke Lemur Center is situated on 80 acres in Duke Forest, Durham NC. Individual animal records have been kept by DLC staff for a total of 4,189 primates owned by the DLC and/or housed at the DLC. Many of the diurnal animals are housed in semi-free-ranging outdoor enclosures that encourage more naturalistic behavior and social interaction. Over the course of the colony’s history, 202 animals were brought in as wild-caught founders. A total of 3,229 animals have been born at the DLC, with an additional 197 individuals DLC-owned but captive born elsewhere (born to DLC-owned dams on loan at other institutions). The remainder is comprised of animals (both wild-born and captive-born) that have transferred into the colony as a loan, donation, or trade from another institution. It is an active breeding colony, so number of animals recorded is continually increasing. Data have been verified in all categories for 3,627 of these individuals, and only those are included in this report.

### Data collection and entry

Animal data have been collected and entered by DLC staff according to standard operating procedures and USDA, AZA, and IACUC guidelines throughout the history of the center (United States Department of Agriculture, Association of Zoos and Aquariums, Institutional Animal Care and Use Committee respectively). Births, deaths, weights, enclosure moves, behaviors, and other significant events are recorded daily by animal care, veterinary, and research staff and subsequently entered into the permanent records by the DLC Registrar. Oldest records are mainly in hand-written or typed paper format, with a move to computerized files as that technology became available. In the mid 1990s, we introduced the use of two databases that provided the ability to link our information with that of other captive facilities through the International Species Information System (ISIS). These databases, the Animal Record Keeping System (ARKS^TM^; ISIS, version 4.0, 2010) and the medical version of ARKS (MedARKS^TM^; ISIS, version 5.54.d, 2011) are still currently in use for data entry, but do not produce analyzable output. We are in the process of migrating to the ISIS-implemented replacement for ARKS (the Zoological Information Management System, ZIMS^TM^; ISIS, version 2.0, 2014) for entry and storage of husbandry data, which will allow us to maintain critical links to ISIS. Additional categories of data not supported by these databases have been entered into spreadsheet format, and the extraction of categorical data from old descriptive text records for transfer to one of the aforementioned storage formats is ongoing. Thus, future updates will contain not only newly collected colony information, but newly extracted and verified information from old and descriptive records as well, and will expand the number of individuals, taxa, and variables available.

### Data extraction, importation, and compilation

The DLC database was built using SAS® software. Data were imported into SAS Enterprise Guide® [SAS, version 4.3 (2010), 5.1(2011)] from the following sources: a) ARKS and MedARKS as.dbf files b) ZIMS as.xml files and c) various categorical data types as Excel files (.csv or.xlsx format). Programs were written in SAS® [SAS, version 9.2 (2009), 9.3 (2011)] to extract, match, and/or join direct variables from the various source files, to perform additional calculations to create new variables, and to format data output. Additional calculations and formatting were carried out using drag and drop tools within SAS Enterprise Guide Projects. Data matching for individual animals is based on a unique DLC identifier (variable name=DLC_ID), while species-related variables are matched based on taxonomic name (variable name=taxon).

### Construction of strepsirrhine life history table

The DLC Life History Table (Table 2 (available online only)) is constructed entirely from variables available in the two associated raw data files provided in this data paper (DLC Animal List and DLC Weight File). Each variable presented in the life history table is named using five terms in the format of Category_Measurement_Group_Variable_Units. The first term is the identifier to indicate a data category as follows: variables relating to sample size or animal counts have an S. Variables relating to reproduction begin with an R. Those relating to body mass have an M, and those relating to longevity and mortality an L. Finally, any variables not relating to those categories begin with an O (other). The second term in the name is the type of measurement, for example N, Mean, Max, Min, Peak or Pct. The third term identifies the group the variable is assessed for: all individuals (All), males only (M) females only (F), individuals of undetermined sex (ND), female parent (Dam), or male parent (Sire). The grouping term is omitted if inappropriate, as is the case for some variables relating to litter size, birth, and breeding season. These three qualifiers are followed by the variable core (e.g., AdultWeight, LitterSize, AgeAtDeath, etc.). Finally a unit of measure is added to the end if needed (y=years, day=days, g=grams). All life history table variable definitions are shown in Table 3 (available online only), and justification and explanation of calculations are given below.

Decisions regarding which individuals and data to include in each summary calculation were guided by our intimate knowledge of captive management and breeding practices, some of which could make certain subsets of data unreliable, and we urge users to consider these limitations as described; additional cautions are given in the ‘Usage Notes’ section below. Should users opt to implement different strategies for generating species summaries, however, they need only to refer to the accompanying data files for the source information. In addition to the reference table provided in this data descriptor, an analytic version of the table is available for direct use in statistical software (see ‘Data Records’ below).

#### Category S: Animal number/sample size variables:

Numbers of animals in various categories were counted to provide sample sizes for specific summary variables presented in the life history table, as well as to provide criteria that researchers can use to determine which species and/or subsets of data may be appropriate for use in other projects based on sample size requirements. Number of individuals in the historic DLC colony includes animals born at the DLC, wild-born animals, animals from other institutions that transferred into the colony at any time in the DLC history, and DLC-owned animals at other institutions (i.e., all animals for which we have data). Numbers of animals in the current DLC colony reflect animals currently living on site at the DLC who are potentially available for research use. Male and female individuals are sexed at birth or acquisition, and in cases where infants were stillborn or died very young and not sexed, sex is designated as ND (not determined). Captive-born (CB) animals were born at the DLC or at another captive facility and have known dates of birth. Wild-born (WB) animals were imported by the DLC or by another institution from the animal’s country of origin and have estimated dates of birth. For some individuals, origin is unknown (U) and they too have estimated dates of birth.

The age of most wild-born animals and animals of unknown origin was estimated on arrival by experienced staff and based on physical appearance, tooth wear, and other morphological characteristics. If the animal’s age at capture was estimated and documented, the date of birth is assigned as follows: for seasonally breeding species, the first day of the middle month of breeding season in the country of origin in the estimated year of birth is used; for non-seasonal breeders, the month and day of acquisition is used with the estimated year of birth.

In some cases an age estimate was not documented and the animal was merely described as ‘adult’. In such cases, the animal was assigned the age equal to the minimum dam age at reproduction for that species (see discussion under ‘reproductive variables’ below) and so any ages calculated for that animal are a minimum. As such, these animals are included in calculations involving the maximum of a variable (the estimated age of an animal cannot be artificially older than the true age) but not the minimum of a variable (estimated age of an animal *could* be artificially younger than the true age). Minimum dam age at conception was used in all determinations of adult age rather than using the dam value for females and the sire value for males. This decision was based on factors of captive breeding management wherein dams are more reliably bred at earliest ages to increase numbers of breeding animals in the colony, but they may be paired with more experienced sires to increase chances of breeding success, making the sire minimum age at conception potentially less reliably accurate as an indicator of adult status than dam minimum age at conception for some species with relatively few breeding sires.

#### Category R: Reproductive variables:

Variables relating to conception (e.g., breeding season, age at conception) use an *expected* date of conception that is calculated by subtracting gestation time from infant date of birth. In an attempt to control for premature births, for which date of conception would be erroneously estimated to be earlier than the true date, infants who did not survive at least one day were excluded from calculations involving date of conception thus excluding any infants who were stillborn due to prematurity.

*Expected gestation* time is assigned for each species based on DLC cases where copulation was observed and documented and the number of days to subsequent infant birth counted. *Gestation time ranges* take into account both these observations and reports of gestation time in wild populations^5–11,5–11,5–11,5–11,5–11,5–11,5–11^. Because much of the information relating to breeding events is still embedded in descriptive records, gestation time variables are a summary rather than a true calculation, with ‘Expected_Gestation_day’ identified as the most commonly used at the DLC for each species, which in most cases lies on the lower end of the gestation range. More thorough analyses of descriptive DLC breeding records may result in fine-tuning of these values in the future, but we have confidence that these are valid estimates because their use with observed breeding behaviors in the current colony predicts birth to within a few days.

Each taxon is characterized as a seasonal (S) or a non-seasonal (NS) breeding species. Birth and Breeding season peaks are based only on infants born at the DLC with known dates of birth and are only calculated for seasonally breeding species. Non-seasonally breeding species show a ‘0’ for these variables. As discussed above, date of conception is calculated by subtracting gestation time from date of birth; date of birth is an exact value, but date of conception is an estimate. We therefore assess seasonal values using month, rather than day, of the event. Peak birth/breeding month is calculated by identifying the month in which the most events occurred for each species; an event is defined here as the birth or conception of a litter, not an individual. Peak birth/breeding season includes sequential months on either side where a) at least one third as many events took place or b) at least 20% of total number of events took place. These constraints were imposed to identify the peak, not necessarily the entire, breeding season. Importantly, their implementation systematically eliminates small tails that may artificially lengthen the true breeding season, especially for species where overall number of births was low and a single early or late pregnancy could extend it.

Minimum ages at reproduction were calculated using only individuals with known dates of birth (i.e., no wild-born animals or animals of unknown origin). Maximum ages at reproduction may include individuals with estimated dates of birth (see discussion above). Litter size variables are based only on animals born at the DLC but do include *all* DLC births (including infants that did not survive 1 day), and include the average, most common, maximum and minimum litter sizes observed for each species, as well as the frequency of the most common litter size. Birth sex ratio of male to female DLC births is calculated for each taxon. Only DLC births are used because animals brought in from the wild or from other institutions may be selected based on programmatic needs (e.g., wild founders were typically imported as male-female pairs) and may mask the underlying birth sex ratio.

#### Category L: Longevity variables:

Maximum age is determined by the age of the oldest individual recorded, living or dead, and includes animals with estimated dates of birth as described above. Longevity was assessed using a proportional hazards model after exclusion of young infant deaths (death prior to 30 days of age) and censoring of living animals and those with uncertain status. Median longevity was derived from the average age of the nearest uncensored values above and below 50% survivorship after each was weighted by distance from the 0.5 midpoint [((Upper age−(Upper age*distance from midpoint))+((Lower age+(Lower age*distance from midpoint)))/2]. Infant mortality percentage is calculated as the percentage of infants born at the DLC who died at less than 30 days of age. Infant mortality here *does* include stillbirths. More thorough analyses of DLC descriptive records will eventually allow us to differentiate between rates of stillbirth and rates of live infant mortality.

#### Category M: Body mass variables:

To eliminate artificially low or high values from unviable stillborn individuals, mean, maximum and minimum neonatal weight calculations include only individuals that survived at least 1 day. Neonatal body mass variables include measurements taken on day 0 (day of birth) or day 1. If both have been recorded for a single individual, the average is used to represent that individual. Inclusion of day 1 weights dramatically increases sample sizes for some species, and individuals variably gain, maintain or lose weight on day 1, so we feel that inclusion of both measurements will yield the most accurate species values. Species values are the mean, maximum, and minimum weights across all individuals of that species.

Adult body mass calculations include all weights obtained after an individual reached *twice* the minimum dam age at reproduction for the species. This age cutoff was enforced in order to ensure that adult body mass values are not artificially lowered due to the inclusion of weights taken during the late ‘near-adult body size’ growth period. An additional category of young-adult body mass was created and includes weights taken when an animal was between minimum age at reproduction and twice the minimum age at reproduction. A comparison of the two categories reveals that young adult averages are indeed lower than the adult averages for 24 of 27 species, indicating that animals of most species are still growing, albeit slowly, during this period. Finally, in order to ensure that weights from wild-caught juveniles of non-exact age did not affect age-based analyses, weights from wild-caught animals at age estimates younger than dam age at first reproduction were excluded.

Mean adult and young adult weights are calculated as follows: weights taken within 60 days of death were excluded, as were weights from pregnant females. If multiple weights were obtained for an individual in a single month, those weights were averaged so as not to bias results from periods of frequent weighing (e.g., for research projects or due to health concerns). The average body weight for each individual was then generated using the mean across all monthly averages for that animal. A mean for all individuals of a species was then calculated from these individual averages. Animals whose average weight was more than two standard deviations above the mean were considered obese (adult=46 of 1,358 individuals; young adult=32 of 932 individuals), and removed from final analyses so as not to skew species means upward^12,13^. The remaining (non-obese) individual means were averaged by species, and by sex where indicated. Maximum and minimum body mass values were obtained from the highest and lowest of these individual averages.

#### Category O: Other variables:

Activity type—nocturnal or diurnal—is indicated for each species (N and D respectively). Numbers of individuals for which biological samples are available in the DLC collection is also shown for each taxon. Biological samples are opportunistically banked, and types of samples available include blood, serum, cadavers, ultra-cold tissues from major and obscure organs, RNA-later infused tissues, and formalin fixed samples. The number and type of samples available for any individual is wildly variable, with some individuals having all of the above sample types available, and others represented by as little as a single eyeball. See below under ‘usage notes/additional data….’ for details of how to obtain more information about samples specific to a species, individual, or sample type.

### Construction of the DLC animal list and weight files

Variables included in the Animal list are single copy variables with a row entry for each of the individuals in the historic DLC colony. The weight file contains multiple copy variables with a row entry for each individual weight measurement recorded. In most cases, the core variables in these files were already being tracked in one of the source systems for current data (e.g., ARKS), and the source was populated with older data extracted from other, inaccessible, formats. Once in categorical formats, the data were compiled as described above. Additional variables were then calculated within the programs that generate the Animal List and Weight File using those core variables of directly entered data. There are core and calculated variables that are overlapping in both files that are included for ease of analysis. Variable definitions and calculations for the DLC Animal List are found in Table 4 (available online only), and for the DLC Weight File in Table 5 (available online only).

## Data Records

### Data record 1a, 1b

The DLC Strepsirrhine Life History Summary Table contains 91 variables for 27 taxa. There are two versions of this file provided; they contain the same information but are formatted for different uses. The first, data record 1a, is a reference version, with all variables in character format in each of 91 rows, and 27 columns each referencing a different taxon. The reference version is designed to facilitate location of particular data points for particular taxon that a user may be interested in. The second, data record 1b, is an analysis version that contains a mixture of character and numeric variables each in one of 91 columns, with a row for each of the 27 species. While it is perhaps more difficult to find a specific data point in this table, this version can be imported directly into analysis software for comparison of variables across taxa. The reference version (1a) can be accessed in the HTML version of this report (Table 2 (available online only)). Data record 1b (Data Citation 1) is stored as comma separated values, and is available from the Dryad Digital Repository. Descriptions of all Life History Table variables can be found in Table 3 (available online only) of this Data Descriptor, and as an associated file in Dryad. The dataset was last updated June 6, 2014.

### Data record 2

**Figure 3:**
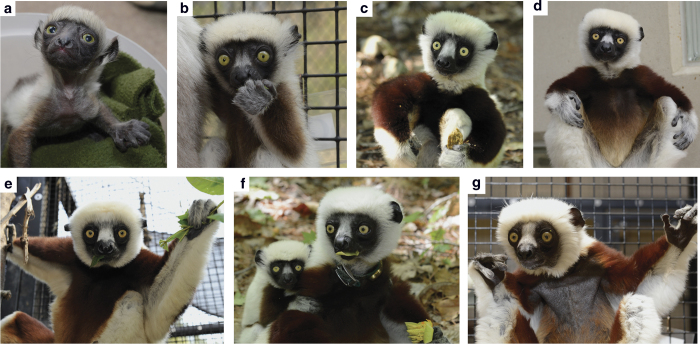
*Propithecus coquereli* life stages. Data files contain information for individuals at all stages of life. Shown are (**a**) newborn (**b**) infant (**c**) juvenile (**d**) yearling (**e**) young adult (**f**) reproductive adult and (**g**) old adult *Propithecus coquereli*.

The DLC Animal List contains 32 variables for 3,627 individuals representing 27 taxa in all stages of life (see Figure 3). This file (Data Citation 1) is stored as comma separated values, and is available from the Dryad Digital Repository. Descriptions of all variables in the Animal List can be found in Table 4 (available online only) of this Data Descriptor, and as an associated file in Dryad. The dataset was last updated June 6, 2014.

### Data record 3

**Figure 4:**
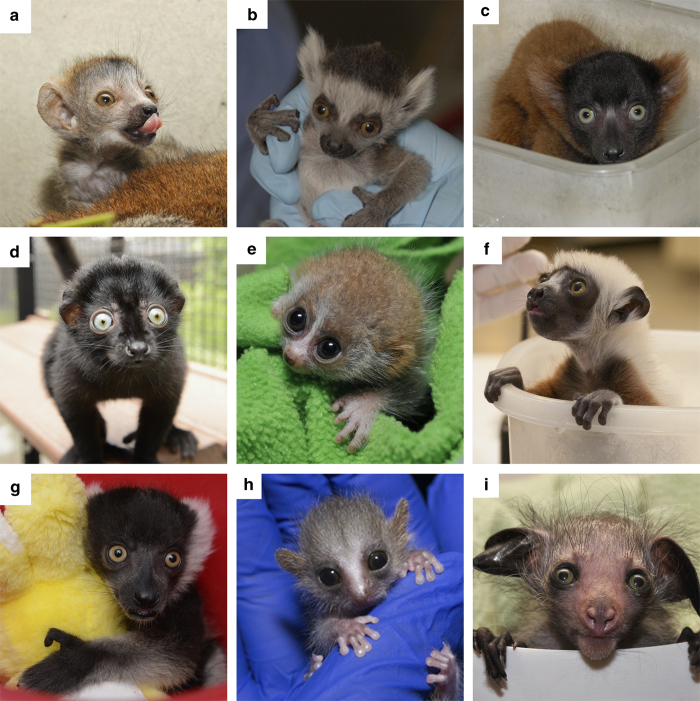
Obtaining strepsirrhine infant weights. Infants are weighed on a regular basis from birth onward so that healthy growth can be monitored. The Weight File contains 14,622 weight values from 1,474 infant and juvenile individuals. Shown are (**a**) *Eulemur coronatus*/crowned lemur (**b**) *Lemur catta*/ring-tailed lemur (**c**) *Varecia rubra*/red ruffed lemur (**d**) *Eulemur flavifrons*/blue-eyed black lemur (**e**) *Nycticebus pygmaeus*/pygmy slow loris (**f**) *Propithecus coquereli*/Coquerel’s sifaka (**g**) *Varecia variegata variegata*/black-and-white ruffed lemur (**h**) *Microcebus murinus*/gray mouse lemur (**i**) *Daubentonia madagascariensis/*aye-aye.

The DLC Weight File contains 33 variables relating to 65,692 weight measurements across 2174 individuals and 27 taxa, and includes a large number of infant and juvenile weights (see Figure 4). This file (Data Citation 1) is stored as comma separated values, and is available from the Dryad Digital Repository. Descriptions of all variables in the Weight File can be found in Table 5 (available online only) of this Data Descriptor, and as an associated file in Dryad. The dataset was last updated June 6, 2014.

## Technical Validation

Once data became accessible and analyzable in the new database, data entries were completed and verified using a variety of methods including 1) identifying missing data and scouring various DLC and ISIS records to find the information if it existed (e.g., identifying missing sires that were actually known but not entered in ARKS) 2) standardizing codes and/or text entries by conducting frequency analyses, double-checking variable types with relatively few entries and correcting typographical errors (e.g., if we find two codes have been used to flag the same type of information, a single code will be selected and the others recoded to match; if we find an misspelled taxon name that yields a single individual, the entry will be corrected), and 3) investigating impossible outliers (e.g., dates that fall outside of the project range, weights that are orders of magnitude out of the proper range, or dates of birth that yield negative calculated ages). In addition, we assessed data output from a series of known individuals to ensure that the output generated by the database programs yielded correct values in all categories. Individuals, taxa, or variables for which verification is not complete are excluded from the data presented here.

## Usage Notes

### Wild vs. captive populations

All data presented are from captive individuals and may not necessarily be representative of wild population values, particularly for some variables. For example, we expect that longevity in captive populations will exceed that of wild populations in most cases due to lack of predation and access to veterinary care in the former. Reproductive variables that are affected by variation in resource availability may also differ as captive populations have continual access to species-appropriate diets with no seasonal or yearly scarcity. Similarly, body mass may be higher in captive populations because they are never resource-challenged. In some cases, the degree to which captive and wild data concur will vary by species and will be affected by sample sizes and methods of analysis. We therefore warn against using these captive-derived values to *directly* assess life history variables in wild populations, but suggest that researchers may be able to use them as *relative indicators* of life history variables in wild populations with caution.

For example, a comparison of body mass variables in this dataset to those for wild populations extracted from the literature for 20 lemur species^14^ reveals that across all species, average adult body masses at the DLC are 19% higher than averages for wild populations summarized from the literature; half of the species are fairly comparable to wild body mass (DLC animals are −1% to +11% heavier), with the other half showing greater discrepancies (DLC animals are +18% to +52% heavier). However, there are some factors that may explain some of the variability. First, in the captive data, we differentiate between adults and young adults, which would have a tendency to increase the DLC average compared to wild estimates from the literature if those contain many young adult individuals. Second, 6 of the 10 more discrepant species are flagged as having ‘large variation in measurements relative to mean body size’ in Table 1 of Taylor and Schwitzer^14^, indicating that those averages may vary seasonally, by population or by study. Finally, the most discrepant species (Eful at 52%) is so distinct from the DLC values that we suspect the possibility that two different subspecies or quite distinct populations have been measured. If all of the cases of questionable data are excluded, the DLC animals are on average 12% heavier than wild populations. Thus, while a fine scale assessment of body size in wild populations cannot be derived from the DLC data, a comparison one species relative to another may provide insightful results.

### Breeding seasonality

All breeding and birth season data are for the captive colony in North America, and are seasonally opposite of breeding and birth seasons in Madagascar, which is home to all wild lemur populations.

### Timing and success of reproduction

There are some analyses that should **not** be conducted with these data because of factors associated with captive breeding management. Breeding is very strictly managed, so *individual* ages at reproduction do not give a true indication of a) individual variation in minimum, maximum, or mean age at reproduction or b) inter-birth interval. If a female does not conceive until well into adulthood, it is much more likely that she wasn’t allowed to breed than that she wasn’t able to breed. Because the sample sizes are large in most cases, we do feel that *species* values for the minimum and maximum ages at reproduction are reliable and as such, they are provided in the life history table.

In the DLC Animal List data file, there is a variable named ‘N_Known_Offspring’ that indicates the number of offspring in our records for that the individual is known to have parented. This should not be taken as a measure of relative reproductive success. In other words, dams with more known offspring are not necessarily more reproductively fit than those with fewer or no known offspring, and it may simply be that the former were given more reproductive opportunities based on management strategies. In addition, some of the offspring counts may be underestimates because a) some individuals may have had offspring at other institutions that are not accounted for in our records, and b) in cases where there are multiple possible sires of an offspring, that offspring is not counted for any of the potential sires.

### Longevity

In some cases with small numbers of uncensored data points, a median longevity could not be calculated because the proportional hazards curve never drops below 50% survivorship. We see this for the aye-aye females, where the oldest female death is still young relative to other data points. As older aye-aye females die over time, these values will be able to be calculated.

### Body mass: seasonal variation

The body mass summaries in the life history table are based on all weights, regardless of season in which they are taken. There is seasonal weight variation in some species, and it is particularly striking in the small nocturnal mouse and dwarf lemurs, where average summer weights (Mmur: Apr-Sept; Cmed: May-Oct) are significantly lower than the average winter weights (Mmur: Oct-March; Cmed: Nov-Apr). If the data are being used for a project that is sensitive to this, users should parcel weight data from the Weight File based on the ‘MonthOfWeight’ variable and use subsets accordingly.

### Hybrid animals

The known *Eulemur* hybrids are a mix of between two and five of the following species: Ealb, Ecol, Eful, Eruf, Esan, and Emac. *Varecia* hybrids are a mix of Vrub and Vvv. Hybrid status is entered in ARKS and in cases where hybrid status is unknown, data output indicates a hybrid status of ‘N’ (not a hybrid). There are 16 animals identified taxonomically as *Eulemur* hybrids that are potentially not hybrids because at least one potential sire is of a species that matches the remainder of the animal’s ancestry (DLC_ID’s 5801, 5802, 5933, 5934, 6087, 5553, 5554, 1574, 2513, 2550, 2551, 3527, 3561, 1554, 6212, 2566). These animals are identifiable in the output because their taxonomic code (Eul) indicates a hybrid animal, but their hybrid status is ‘N’. ZIMS *can* produce output indicating ‘hybrid status unknown’, so these entries will be adjusted once the migration from ARKS to ZIMS is complete.

### Additional data, updates, and project information

Interested users may obtain additional access to these data files and future updated versions of data or other DLC project information as follows:

#### 1) Direct download

As outlined above, data records are available in Dryad. Users may also download data files by connecting to the Duke Lemur Center web site (http://lemur.duke.edu/discover/for-researchers/) and navigating to the ‘historic animal data’ page where they will be guided through a brief registration (free) and then be connected to the download page. There are no costs associated with these data downloads. The download page is where future updated versions of the data described here will reside, and updates will also be deposited in Dryad on a yearly basis.

#### 2) Specified data file requests

To inquire about or request a specific dataset that may include information not presented here, please contact corresponding author and DLC data manager, Sarah Zehr, at sarah.zehr@duke.edu. There is currently no cost for such requests, but fees may be implemented in the future to offset the cost of database maintenance.

#### 3) Live animal or biological sample projects

For more information about use of the live DLC colony or acquisition of biological samples, please go to the Duke Lemur Center web site (http://lemur.duke.edu) and navigate to the research page to find more information about these classes of research, as well as contact information for the DLC Research Manager who oversees them. There are fees associated with both live animal use and biological sample purchase.

## Additional information

**How to cite this article:** Zehr, S. M. *et al.* Life history profiles for 27 strepsirrhine primate taxa generated using captive data from the Duke Lemur Center. *Sci. Data* 1:140019 doi: 10.1038/sdata.2014.19 (2014).

## Supplementary Material



## Figures and Tables

**Table 1:** List of taxa included in the data files, including taxonomic code used in all data files (Taxon), Latin name and common name of each taxon^4,5^.

**count**	**Taxon**	**Latin_Name**	**Common_Name**
1	CMED	*Cheirogaleus medius*	Fat-tailed dwarf lemur
2	DMAD	*Daubentonia madagascariensis*	Aye-aye
3	EALB	*Eulemur albifrons*	White-fronted brown lemur
4	ECOL	*Eulemur collaris*	Collared brown lemur
5	ECOR	*Eulemur coronatus*	Crowned lemur
6	EFLA	*Eulemur flavifrons*	Blue-eyed black lemur
7	EFUL	*Eulemur fulvus*	Common brown lemur
8	EMAC	*Eulemur macaco*	Black lemur
9	EMON	*Eulemur mongoz*	Mongoose lemur
10	ERUB	*Eulemur rubriventer*	Red-bellied lemur
11	ERUF	*Eulemur rufus*	Red-fronted brown lemur
12	ESAN	*Eulemur sanfordi*	Sanford's brown lemur
13	EUL	*Eulemur*	Eulemur hybrid
14	GMOH	*Galago moholi*	Mohol bushbaby
15	HGG	*Hapalemur griseus griseus*	Eastern lesser bamboo lemur
16	LCAT	*Lemur catta*	Ring-tailed lemur
17	LTAR	*Loris tardigradus*	Slender loris
18	MMUR	*Mircocebus murinus*	Gray mouse lemur
19	MZAZ	*Mirza coquereli*	Northern giant mouse lemur
20	NCOU	*Nycticebus coucang*	Slow loris
21	NPYG	*Nycticebus pygmaeus*	Pygmy slow loris
22	OGG	*Otolemur garnettii garnettii*	Northern greater galago
23	PCOQ	*Propithecus coquereli*	Coquerel's sifaka
24	PPOT	*Perodicticus potto*	Potto
25	VAR	*Varecia*	Varecia hybrid
26	VRUB	*Varecia rubra*	Red ruffed lemur
27	VVV	*Varecia variegata variegata*	Black-and-white ruffed lemur

**Table 2:** DLC Strepsirrhine Life History Table

**Variable**	**CMED**	**DMAD**	**EALB**	**ECOL**	**ECOR**	**EFLA**	**EFUL**	**EMAC**	**EMON**	**ERUB**	**ERUF**	**ESAN**	**EUL**	**GMOH**	**HGG**	**LCAT**	**LTAR**	**MMUR**	**MZAZ**	**NCOU**	**NPYG**	**OGG**	**PCOQ**	**PPOT**	**VAR**	**VRUB**	**VVV**
S_N_All_Historic	252	44	40	71	77	75	65	119	128	30	170	22	274	324	62	330	61	277	110	85	49	339	176	33	50	173	191
S_N_M_Historic	122	22	20	38	44	39	37	58	61	18	77	11	135	152	27	155	26	141	45	40	23	167	94	14	18	80	102
S_N_F_Historic	99	22	18	31	29	35	25	53	65	12	86	11	133	136	35	157	27	122	52	44	23	142	76	13	31	86	81
S_N_ND_Historic	31	.	2	2	4	1	3	8	2	.	7	.	6	36	.	18	8	14	13	1	3	30	6	6	1	7	8
S_N_All_CaptiveBorn	248	37	32	65	72	68	58	114	106	24	163	17	273	265	48	326	54	260	104	59	38	293	160	20	50	163	182
S_N_M_CaptiveBorn	120	19	16	34	42	37	33	56	51	15	73	9	135	127	22	153	23	137	42	30	18	145	87	9	18	75	99
S_N_F_CaptiveBorn	97	18	14	29	26	30	22	50	53	9	83	8	132	102	26	155	23	109	49	28	17	118	67	5	31	81	75
S_N_All_WildBorn	4	7	6	6	4	7	6	5	18	6	7	5	.	44	14	2	7	17	6	17	11	17	16	10	.	9	9
S_N_M_WildBorn	2	3	3	4	2	2	3	2	8	3	4	2	.	16	5	1	3	4	3	8	5	9	7	4	.	4	3
S_N_F_WildBorn	2	4	3	2	2	5	3	3	10	3	3	3	.	28	9	1	4	13	3	9	6	8	9	6	.	5	6
S_N_All_CurrentResident	19	16	1	4	10	15	.	3	13	6	2	1	4	.	3	35	1	48	.	1	7	.	31	.	.	15	10
S_N_M_CurrentResident	11	6	.	2	5	11	.	1	7	2	1	.	3	.	1	15	.	24	.	.	4	.	15	.	.	7	7
S_N_F_CurrentResident	8	10	1	2	4	4	.	2	6	4	1	1	1	.	2	20	1	24	.	1	3	.	16	.	.	8	3
S_N_All_DLCBorn_Infant	226	28	28	59	61	57	44	99	80	22	144	17	245	251	46	297	50	225	93	43	31	234	108	16	49	131	158
S_N_M_DLCBorn_Infant	110	12	13	32	35	29	50	25	41	13	65	9	120	121	20	134	20	114	36	19	17	117	56	8	18	61	84
S_N_F_DLCBorn_Infant	87	16	13	25	22	28	16	42	38	9	75	8	121	94	26	145	22	97	44	23	11	87	48	2	30	65	66
S_N_ND_DLCBorn_Infant	29	.	2	2	4	.	3	7	1	.	4	.	4	36	.	18	8	14	13	1	3	30	4	6	1	5	8
R_Ratio_MtoF_DLCBirths	1.264	0.750	1.000	1.280	1.591	1.036	1.563	1.190	1.079	1.444	0.867	1.125	0.992	1.287	0.769	0.924	0.909	1.175	0.818	0.826	1.545	1.345	1.167	4.000	0.600	0.938	1.273
S_N_All_DLCBorn_Litter	102	28	17	48	49	55	39	72	78	21	133	16	209	174	42	229	49	97	59	43	18	212	108	16	24	59	83
R_Mean_LitterSize	2.22	1.00	1.65	1.23	1.24	1.04	1.13	1.38	1.03	1.05	1.08	1.06	1.17	1.44	1.10	1.30	1.02	2.32	1.58	1.00	1.72	1.10	1.00	1.00	2.04	2.22	1.90
R_MostCommon_LitterSize	2	1	1,2	1	1	1	1	1	1	1	1	1	1	1	1	1	1	3	2	1	2	1	1	1	2	2,3	2
R_Freq_MostCommon_LitterSize	0.40	1.00	0.47	0.77	0.76	0.96	0.87	0.63	0.97	0.95	0.92	0.94	0.83	0.56	0.90	0.71	0.98	0.39	0.58	1.00	0.61	0.90	1.00	1.00	0.46	0.37	0.43
R_Min_LitterSize	1	1	1	1	1	1	1	1	1	1	1	1	1	1	1	1	1	1	1	1	1	1	1	1	1	1	1
R_Max_LitterSize	5	1	4	2	2	2	2	2	2	2	2	2	3	2	2	3	2	4	2	1	3	2	1	1	3	4	4
R_Expected_Gestation_d	60	165	120	120	120	120	120	120	120	120	120	120	120	124	145	130	167	60	90	193	185	129	160	170	98	98	98
R_Range_Gestation_d	60–64	157–172	120–128	120–128	120–126	120–129	120–128	120–129	120–128	120–127	120–128	120–128	120–128	110–126	145–150	130–136	150–169	57–63	89–90	185–197	183–198	126–134	155–168	170–172	98–102	98–102	98–102
R_Pattern_Breeding	S	NS	S	S	S	S	S	S	S	S	S	S	S	NS	S	S	NS	S	NS	NS	NS	NS	S	NS	S	S	S
R_Peak_Breeding_Month	5	0	12	12	1	11	12	11	12	12	11	1	12	0	12	11	0	4	0	0	0	0	9	0	1	1	1
R_Peak_Breeding_Season	4.5.6	0	1.12	1.11.12	1.12	11	11.12	11.12	1.12	1.11.12	11.12	1.3.12	1.11.12	0	11.12	11	0	4.5.6.7	0	0	0	0	7.8.9	0	1.2.12	1.2	1.2.12
R_Peak_Birth_Month	7	0	4	4	5	3	4	3	4	4	3	5	4	0	5	3	0	6	0	0	0	0	2	0	4	4	4
R_Peak_Birth_Season	6.7.8	0	4.5	3.4.5	4.5	3	3.4	3.4	4.5	3.4.5	3.4	4.5.6.7	3.4.5	0	4.5.6	3.4	0	6.7.8	0	0	0	0	1.2.3.12	0	4.5	4.5	4.5
R_Min_Dam_AgeAtConcep_y	0.80	4.22	2.57	1.64	1.71	1.59	1.39	1.48	1.78	1.78	1.55	1.61	1.01	0.40	1.53	1.34	0.99	0.60	0.82	1.33	1.87	0.59	2.64	2.16	1.64	1.67	1.61
R_Min_Sire_AgeAtConcep_y	1.80	3.66	2.75	2.52	2.48	2.66	1.74	0.73	2.92	3.70	1.50	2.03	1.60	1.02	3.70	1.66	1.04	0.60	1.41	1.28	3.74	0.76	2.60	3.15	.	1.79	2.68
R_Max_Dam_AgeAtConcep_y	14.85	26.03	11.31	23.20	17.47	16.89	19.18	15.69	23.60	13.15	22.37	19.28	17.70	8.48	16.98	21.70	13.38	13.32	12.84	8.16	11.35	14.65	20.40	17.63	6.73	24.26	23.41
R_Max_Sire_AgeAtConcep_y	16.11	28.47	16.31	21.22	19.92	21.13	19.18	14.73	19.69	14.30	24.29	8.43	9.65	6.63	15.98	29.53	11.63	9.47	13.70	14.86	11.56	10.84	29.76	13.63	.	21.79	31.84
R_Active_DLCBreeding	Y	Y	N	N	Y	Y	N	N	Y	N	N	N	N	N	N	Y	N	Y	N	N	Y	N	Y	N	N	Y	Y
S_N_All_AdultsWeighed	116	16	8	38	34	41	24	37	58	17	47	17	62	21	30	120	29	158	65	51	21	66	44	13	6	74	49
M_Mean_All_AdultWeight_g	240.96	2669.20	2233.24	2336.89	1645.08	2437.08	2485.50	2467.48	1554.81	2191.48	2285.43	2111.87	2417.95	169.25	1012.49	2454.66	185.64	82.65	302.41	1168.49	487.02	1059.21	3982.55	919.86	3336.21	3601.30	3492.32
M_Min_All_AdultWeight_g	144.07	2265.00	1840.43	1697.22	1220.19	2180.44	1670.00	2084.88	1301.03	1770.00	1620.00	1840.00	1793.91	140.83	794.08	1873.49	152.00	56.50	250.68	807.67	357.20	729.00	3003.17	768.74	2815.00	2710.50	2903.25
M_Max_All_AdultWeight_g	338.50	2987.24	2606.00	2735.00	1993.00	2820.41	3076.33	2867.67	1920.00	2506.27	2897.00	2493.00	3360.00	201.18	1292.87	3049.16	220.50	118.00	357.88	1506.07	612.67	1335.00	4939.49	1139.50	3913.00	4200.00	4206.49
S_N_M_AdultsWeighed	67	7	6	24	18	25	14	18	30	9	24	8	29	10	13	66	14	85	32	27	9	34	24	8	2	32	27
M_Mean_M_AdultWeight_g	236.31	2603.15	2155.01	2318.30	1698.05	2382.84	2366.78	2387.38	1524.53	2050.08	2262.57	2039.70	2388.04	179.38	1022.57	2543.22	185.72	79.82	298.30	1144.36	495.96	1137.08	3896.12	943.99	3364.00	3586.68	3450.73
M_Min_M_AdultWeight_g	144.07	2265.00	1840.43	1697.22	1220.19	2180.44	1670.00	2084.88	1301.03	1770.00	1745.45	1840.00	1820.50	149.70	910.99	2092.68	152.00	56.50	250.68	807.67	405.56	908.67	3233.03	768.74	2815.00	3128.50	2903.25
M_Max_M_AdultWeight_g	337.00	2884.86	2606.00	2735.00	1993.00	2750.84	3066.25	2691.29	1818.19	2278.64	2897.00	2435.50	3095.50	201.18	1240.00	3049.16	219.65	112.50	345.50	1506.07	556.86	1304.13	4751.31	1139.50	3913.00	4200.00	4129.29
S_N_F_AdultsWeighed	49	9	2	14	16	16	10	19	28	8	23	9	33	11	17	54	15	73	33	24	12	32	20	5	4	42	22
M_Mean_F_AdultWeight_g	247.33	2720.57	2467.94	2368.77	1585.49	2521.82	2651.71	2543.36	1587.27	2350.55	2309.28	2176.03	2444.23	160.04	1004.77	2346.42	185.57	85.93	306.41	1195.63	480.31	976.48	4086.26	881.26	3322.31	3612.43	3543.37
M_Min_F_AdultWeight_g	191.86	2329.71	2348.39	1930.97	1308.30	2205.00	2261.00	2120.00	1386.71	2263.02	1620.00	1963.40	1793.91	140.83	794.08	1873.49	160.61	57.00	261.73	856.75	357.20	729.00	3003.17	777.96	3154.25	2710.50	2935.33
M_Max_F_AdultWeight_g	338.50	2987.24	2587.50	2679.03	1927.50	2820.41	3076.33	2867.67	1920.00	2506.27	2801.00	2493.00	3360.00	170.26	1292.87	2935.50	220.50	118.00	357.88	1489.83	612.67	1335.00	4939.49	1118.00	3529.00	4152.96	4206.49
S_N_All_NeonatesWeighed	16	24	.	4	3	10	4	5	10	3	11	1	11	16	7	47	6	7	5	16	15	19	54	2	3	24	21
M_Mean_All_NeonateWeight_g	13.41	109.93	.	68.48	48.07	82.65	73.00	70.10	62.28	76.53	77.06	93.60	87.73	12.37	50.40	65.08	11.53	8.11	16.36	51.18	25.70	48.59	106.86	38.30	120.00	109.73	107.17
M_Min_All_NeonateWeight_g	8.00	68.50	.	27.80	45.20	55.50	36.00	60.00	51.50	70.10	43.00	93.60	70.00	7.70	46.00	34.50	8.00	5.40	13.70	40.00	16.50	40.00	84.50	33.00	98.00	68.00	68.00
M_Max_All_NeonateWeight_g	21.00	136.00	.	99.10	51.00	102.50	92.00	83.00	77.50	89.00	94.00	93.60	112.00	19.00	57.00	90.00	14.50	12.70	19.90	65.00	32.25	56.50	131.50	43.60	139.00	135.50	137.00
S_N_M_NeonatesWeighed	11	10	.	2	1	5	2	4	4	2	4	1	5	8	3	20	2	4	2	7	11	12	25	2	.	10	17
M_Mean_M_NeonateWeight_g	13.00	111.37	.	86.55	51.00	76.42	60.50	68.50	63.13	70.30	88.13	93.60	90.80	12.73	48.60	64.21	12.25	7.18	15.10	50.64	24.52	48.94	107.03	38.30	.	108.15	106.18
M_Min_M_NeonateWeight_g	8.00	100.50	.	74.00	51.00	55.50	36.00	60.00	51.50	70.10	83.00	93.60	77.00	7.70	47.80	34.50	10.00	5.50	13.70	40.00	16.50	41.40	84.50	33.00	.	88.00	68.00
M_Max_M_NeonateWeight_g	21.00	136.00	.	99.10	51.00	90.60	85.00	83.00	77.50	70.50	94.00	93.60	102.00	19.00	50.00	90.00	14.50	9.00	16.50	62.50	30.50	56.50	124.00	43.60	.	128.00	129.50
S_N_F_NeonatesWeighed	5	14	.	2	2	5	2	1	6	1	7	.	6	8	4	27	4	3	3	9	4	7	29	.	3	14	4
M_Mean_F_NeonateWeight_g	14.32	108.90	.	50.40	46.60	88.88	85.50	76.50	61.72	89.00	70.74	.	85.17	12.01	51.75	65.72	11.18	9.37	17.20	51.59	28.94	47.99	106.72	.	120.00	110.86	111.38
M_Min_F_NeonateWeight_g	10.50	68.50	.	27.80	45.20	73.70	79.00	76.50	54.00	89.00	43.00	.	70.00	7.80	46.00	40.00	8.00	5.40	15.20	41.00	25.00	40.00	92.00	.	98.00	68.00	88.00
M_Max_F_NeonateWeight_g	18.00	131.50	.	73.00	48.00	102.50	92.00	76.50	66.60	89.00	86.50	.	112.00	14.70	57.00	89.00	14.00	12.70	19.90	65.00	32.25	55.50	131.50	.	139.00	135.50	137.00
S_N_All_YngAdltsWeighed	55	23	7	29	21	37	11	28	48	14	28	14	30	12	26	109	25	65	25	28	27	31	56	6	9	81	48
M_Mean_All_YngAdultWeight_g	241.71	2575.28	2170.29	2282.38	1575.58	2256.35	2222.89	2366.93	1493.68	2004.27	2063.73	1941.25	2098.08	143.38	1009.71	2116.96	175.10	67.66	288.53	1134.81	503.35	867.16	3972.20	853.07	3346.56	3432.81	3185.20
M_Min_All_YngAdultWeight_g	128.33	1771.67	1948.38	1741.83	1212.00	1963.63	1843.50	1966.00	1220.00	1750.00	1682.25	1720.00	1465.00	123.60	697.00	1483.00	146.50	51.92	247.00	858.57	350.52	674.00	3141.14	733.46	2746.00	2623.91	2459.50
M_Max_All_YngAdultWeight_g	337.00	2852.14	2528.50	2747.67	1927.50	2627.14	2629.00	2873.50	1741.25	2220.00	2515.00	2195.00	2702.00	165.67	1237.50	2653.00	219.83	95.00	340.00	1500.50	683.52	1087.97	5150.33	1039.00	3609.00	4190.50	3868.00
S_N_M_YngAdultsWeighed	23	12	6	17	12	21	7	17	23	7	15	7	16	5	9	50	14	35	14	14	16	18	27	5	1	37	32
M_Mean_M_YngAdultWeight_g	242.84	2501.20	2193.67	2291.63	1597.23	2210.81	2192.79	2284.03	1484.79	1958.17	2063.02	1948.24	2080.02	149.82	1005.79	2128.46	180.76	67.03	284.74	1151.70	496.53	919.18	3791.60	867.51	3562.00	3437.41	3182.48
M_Min_M_YngAdultWeight_g	128.33	1771.67	1948.38	1928.50	1343.33	2007.00	1843.50	1966.00	1220.00	1750.00	1687.25	1720.00	1465.00	133.75	697.00	1600.00	160.33	51.92	247.00	858.57	350.69	736.00	3141.14	733.46	3562.00	2792.00	2459.50
M_Max_M_YngAdultWeight_g	334.00	2821.30	2528.50	2669.00	1927.50	2463.33	2551.00	2796.00	1700.50	2214.50	2452.00	2195.00	2702.00	165.67	1156.33	2611.00	219.83	88.00	326.65	1500.50	589.53	1087.97	4389.67	1039.00	3562.00	4190.50	3868.00
S_N_F_YngAdultsWeighed	32	11	1	12	9	16	4	11	25	7	13	7	14	7	17	59	11	30	11	14	11	13	29	1	8	44	16
M_Mean_F_YngAdultWeight_g	240.90	2656.10	2030.00	2269.28	1546.71	2316.13	2275.56	2495.06	1501.85	2050.36	2064.55	1934.26	2118.73	138.78	1011.79	2107.22	167.90	68.41	293.35	1117.91	513.27	795.13	4140.35	780.91	3319.63	3428.93	3190.65
M_Min_F_YngAdultWeight_g	159.00	2446.25	2030.00	1741.83	1212.00	1963.63	1870.00	2060.00	1264.00	1880.00	1682.25	1796.00	1503.00	123.60	836.00	1483.00	146.50	54.64	248.00	909.94	350.52	674.00	3562.89	780.91	2746.00	2623.91	2569.00
M_Max_F_YngAdultWeight_g	337.00	2852.14	2030.00	2747.67	1796.17	2627.14	2629.00	2873.50	1741.25	2220.00	2515.00	2098.83	2669.00	158.00	1237.50	2653.00	208.50	95.00	340.00	1386.50	683.52	948.87	5150.33	780.91	3609.00	4110.00	3580.50
L_Max_All_Age_y	28.99	32.37	34.07	32.61	27.42	24.37	36.00	30.23	34.86	31.70	32.18	31.70	29.38	16.64	23.40	31.08	21.54	17.96	19.97	24.04	19.26	20.01	30.59	32.35	16.67	37.54	39.39
L_Max_M_Age_y	28.99	32.37	27.75	32.11	26.24	24.26	34.45	30.23	34.86	31.70	32.16	24.19	28.83	16.64	23.03	31.08	21.54	17.96	18.06	22.18	15.51	20.01	30.59	25.67	16.67	29.91	39.39
L_Max_F_Age_y	25.18	30.53	34.07	32.61	27.42	24.37	36.00	30.21	28.07	29.70	32.18	31.70	29.38	14.45	23.40	26.38	18.64	15.94	19.97	24.04	19.26	18.91	21.69	32.35	14.13	37.54	35.68
L_Median_All_Longevity_gt30d_y	14.04	24.05	17.90	20.71	18.14	18.33	18.38	13.77	25.11	16.77	20.16	20.03	17.07	6.82	10.45	16.87	13.38	8.35	13.00	11.26	11.25	12.73	10.36	15.04	7.14	20.68	15.24
L_Median_M_Longevity_gt30d_y	13.05	23.50	15.31	20.72	17.98	17.96	17.77	13.05	24.92	14.65	20.59	18.12	16.51	6.73	10.12	17.63	13.92	8.50	12.70	12.01	10.44	12.64	10.89	14.36	9.24	20.77	15.44
L_Median_F_Longevity_gt30d_y	16.64	.	23.65	19.71	17.36	17.98	20.65	14.71	21.99	.	19.60	20.33	16.80	6.77	11.24	14.89	12.35	8.32	12.46	11.01	13.32	12.35	9.11	13.99	6.53	20.45	14.97
S_N_All_Survival_gt30d	183	40	34	55	54	56	53	87	107	22	133	21	219	230	47	271	44	240	77	78	36	262	139	22	41	139	146
S_N_M_Survival_gt30d	104	20	19	31	31	31	33	47	53	13	60	10	113	114	20	134	23	124	38	37	18	134	75	10	16	62	84
S_N_F_Survival_gt30d	79	20	15	24	23	25	20	39	54	9	70	11	105	115	27	137	21	116	39	41	18	128	63	12	25	77	60
L_Pct_All_InfMort_lt30d	0.30	0.11	0.21	0.25	0.38	0.28	0.27	0.32	0.24	0.36	0.26	0.06	0.21	0.37	0.33	0.20	0.34	0.16	0.35	0.14	0.42	0.33	0.24	0.69	0.18	0.16	0.28
L_Pct_M_InfMort_lt30d	0.16	0.08	0.08	0.22	0.37	0.21	0.16	0.22	0.20	0.38	0.26	0.11	0.18	0.31	0.35	0.16	0.15	0.13	0.19	0.11	0.29	0.28	0.27	0.50	0.11	0.18	0.21
L_Pct_F_InfMort_lt30d	0.23	0.13	0.23	0.24	0.27	0.36	0.31	0.33	0.26	0.33	0.21	.	0.21	0.22	0.31	0.14	0.27	0.06	0.30	0.13	0.45	0.16	0.15	0.50	0.20	0.08	0.32
L_Pct_ND_InfMort_lt30d	1.00	.	1.00	1.00	1.00	.	1.00	1.00	1.00	.	1.00	.	1.00	0.97	.	1.00	1.00	1.00	1.00	1.00	1.00	1.00	1.00	1.00	1.00	1.00	0.75
S_N_All_InfMort_lt30d	67	3	6	15	23	16	12	32	19	8	37	1	51	94	15	59	17	35	33	6	13	77	26	11	9	21	45
S_N_M_InfMort_lt30d	18	1	1	7	13	6	4	11	8	5	17	1	21	38	7	21	3	15	7	2	5	33	15	4	2	11	18
S_N_F_InfMort_lt30d	20	2	3	6	6	10	5	14	10	3	16	.	26	21	8	20	6	6	13	3	5	14	7	1	6	5	21
S_N_ND_InfMort_lt30d	29	.	2	2	4	.	3	7	1	.	4	.	4	35	.	18	8	14	13	1	3	30	4	6	1	5	6
O_NocturnalOrDiurnal	N	N	D	D	D	D	D	D	D	D	D	D	D	N	D	D	N	N	N	N	N	N	D	N	D	D	D
O_N_All_Biosample_Individuals	43	34	5	35	34	.	7	.	48	13	32	11	41	57	34	122	19	46	28	24	21	27	85	7	2	53	43

**Table 3:** DLC Strepsirrhine Life History Table variable definitions

**count**	**Variable Name**	**Variable Definition**
1	S_N_All_Historic	# of individuals recorded in DLC historic colony
2	S_N_M_Historic	# of males recorded in DLC historic colony
3	S_N_F_Historic	# of females recorded in DLC historic colony
4	S_N_ND_Historic	# of individuals recorded in DLC historic colony whose sex was not determined
5	S_N_All_CaptiveBorn	# of captive-born individuals in DLC historic colony
6	S_N_M_CaptiveBorn	# of captive-born males in DLC historic colony
7	S_N_F_CaptiveBorn	# of captive born females in DLC historic colony
8	S_N_All_WildBorn	# of wild-born individuals in DLC historic colony
9	S_N_M_WildBorn	# of wild-born males in DLC historic colony
10	S_N_F_WildBorn	# of wild-born females in DLC historic colony
11	S_N_All_CurrentResident	# of individuals in DLC current colony
12	S_N_M_CurrentResident	# of males in DLC current colony
13	S_N_F_CurrentResident	# of females in DLC current colony
14	S_N_All_DLCBorn_Infant	# of individuals born at DLC
15	S_N_M_DLCBorn_Infant	# of males born at DLC
16	S_N_F_DLCBorn_Infant	# of females born at DLC
17	S_N_ND_DLCBorn_Infant	# of individuals born at DLC whose sex was not determined
18	R_Ratio_MtoF_DLCBirths	Birth sex ratio (#males:1 female) among DLC births.
19	S_N_All_DLCBorn_Litter	# of litters born at DLC
20	R_Mean_LitterSize	Mean litter size
21	R_MostCommon_LitterSize	Most frequent litter size
22	R_Freq_MostCommon_LitterSize	Frequency of the most common litter size
23	R_Min_LitterSize	Minimum litter size
24	R_Max_LitterSize	Maximum litter size
25	R_Expected_Gestation_d	Expected gestation length based on DLC observations and reports in the literature, in days
26	R_Range_Gestation_d	Range of gestation times based on DLC observations and reports in the literature, in days
27	R_Pattern_Breeding	Whether the species breeds seasonally (S) or non-seasonally (NS).
28	R_Peak_Breeding_Month	Peak breeding month
29	R_Peak_Breeding_Season	Peak breeding season
30	R_Peak_Birth_Month	Peak birth month
31	R_Peak_Birth_Season	Peak birth season
32	R_Min_Dam_AgeAtConcep_y	Minimum dam age at conception in years
33	R_Min_Sire_AgeAtConcep_y	Minimum sire age at conception in years
34	R_Max_Dam_AgeAtConcep_y	Maximum dam age at conception in years
35	R_Max_Sire_AgeAtConcep_y	Maximum sire age at conception in years
36	R_Active_DLCBreeding	Whether or not the species is actively breeding in the DLC current colony (Y=yes; N=no)
37	S_N_All_AdultsWeighed	# of adults used to calculate adult body mass; adult defined as: age >= 2x Dam_Min_AAC
38	M_Mean_All_AdultWeight_g	Mean adult body mass, both sexes combined
39	M_Min_All_AdultWeight_g	Minimum adult body mass, both sexes combined
40	M_Max_All_AdultWeight_g	Maximum adult body mass, both sexes combined
41	S_N_M_AdultsWeighed	# of males used to calculate male adult body mass
42	M_Mean_M_AdultWeight_g	Mean adult body mass of males
43	M_Min_M_AdultWeight_g	Minimum adult body mass of males
44	M_Max_M_AdultWeight_g	Maximum adult body mass of males
45	S_N_F_AdultsWeighed	# of females used to calculate female adult body mass
46	M_Mean_F_AdultWeight_g	Mean adult body mass of females
47	M_Min_F_AdultWeight_g	Minimum adult body mass of females
48	M_Max_F_AdultWeight_g	Maximum adult body mass of females
49	S_N_All_NeonatesWeighed	# of neonates used to calculate neonate body mass; neonate defined as: age=DOB or DOB+1 and survived at least 1 day.
50	M_Mean_All_NeonateWeight_g	Mean neonate body mass, both sexes combined
51	M_Min_All_NeonateWeight_g	Minimum neonate body mass, both sexes combined
52	M_Max_All_NeonateWeight_g	Maximum neonate body mass, both sexes combined
53	S_N_M_NeonatesWeighed	# of males used to calculate male neonate body mass
54	M_Mean_M_NeonateWeight_g	Mean neonate body mass of males
55	M_Min_M_NeonateWeight_g	Minimum neonate body mass of males
56	M_Max_M_NeonateWeight_g	Maximum neonate body mass of males
57	S_N_F_NeonatesWeighed	# of females used to calculate female neonate body mass
58	M_Mean_F_NeonateWeight_g	Mean neonate body mass of females
59	M_Min_F_NeonateWeight_g	Minimum neonate body mass of females
60	M_Max_F_NeonateWeight_g	Maximum neonate body mass of females
61	S_N_All_YngAdltsWeighed	# of young adults used to calculate young adult body mass; young adult defined as: Dam_Min_AAC <= age<2x Dam_Min_AAC
62	M_Mean_All_YngAdultWeight_g	Mean young adult body mass, both sexes combined
63	M_Min_All_YngAdultWeight_g	Minimum young adult body mass, both sexes combined
64	M_Max_All_YngAdultWeight_g	Maximum young adult body mass, both sexes combined
65	S_N_M_YngAdultsWeighed	# of males used to calculate male young adult body mass
66	M_Mean_M_YngAdultWeight_g	Mean young adult body mass of males
67	M_Min_M_YngAdultWeight_g	Minimum young adult body mass of males
68	M_Max_M_YngAdultWeight_g	Maximum young adult body mass of males
69	S_N_F_YngAdultsWeighed	# of females used to calculate female young adult body mass
70	M_Mean_F_YngAdultWeight_g	Mean young adult body mass of females
71	M_Min_F_YngAdultWeight_g	Minimum young adult body mass of females
72	M_Max_F_YngAdultWeight_g	Maximum young adult body mass of females
73	L_Max_All_Age_y	Maximum age attained by any individual, living or dead
74	L_Max_M_Age_y	Maximum age attained by any male, living or dead
75	L_Max_F_Age_y	Maximum age attained by any female, living or dead
76	L_Median_All_Longevity_gt30d_y	Median longevity of individuals that survived at least 30 days
77	L_Median_M_Longevity_gt30d_y	Median longevity of males that survived at least 30 days
78	L_Median_F_Longevity_gt30d_y	Median longevity of females that survived at least 30 days
79	S_N_All_Survival_gt30d	# of all individuals that survived at least 30 days
80	S_N_M_Survival_gt30d	# of males that survived at least 30 days
81	S_N_F_Survival_gt30d	# of females that survived at least 30 days
82	L_Pct_All_InfMort_lt30d	% of individuals born at the DLC that did not suvive to 30 days
83	L_Pct_M_InfMort_lt30d	% of males born at the DLC that did not survive to 30 days
84	L_Pct_F_InfMort_lt30d	% of females born at the DLC that that did not survive to 30 days
85	L_Pct_ND_InfMort_lt30d	% of individuals where sex was not determined born at the DLC that did not survive to 30 days
86	S_N_All_InfMort_lt30d	# of individuals born at the DLC that did not suvive to 30 days
87	S_N_M_InfMort_lt30d	# of males born at the DLC that did not survive to 30 days
88	S_N_F_InfMort_lt30d	# of females born at the DLC that that did not survive to 30 days
89	S_N_ND_InfMort_lt30d	# of individuals where sex was not determined born at the DLC that did not survive to 30 days
90	O_NocturnalOrDiurnal	N=Nocturnal species; D=Diurnal species
91	O_N_All_Biosample_Individuals	# of individuals for which biological samples have been banked for research use
See Methods section for details and justification for each variable.		

**Table 4:** DLC Animal List variable descriptions

**count**	**Animal List variable name**	**Animal List Variable Definition**
1	Taxon	Taxonomic code: In most cases, comprised of the first letter of the genus and the first three letters of the species; if taxonomic designation is a subspecies, comprised of the first letter of genus, species, and subspecies, and hybrids are indicated by the first three letters of the genus. See Table 1 for details.
2	DLC_ID	Specimen ID: Unique identification number assigned by the DLC at accession of animal.
3	Hybrid	Hybrid status: N=not a hybrid. S=species hybrid. B=subspecies hybrid. If sire is one of multiple possible and animal could be a hybrid, it is designated a hybrid.
4	Sex	Sex: M=male. F=Female. ND=Not determined
5	Name	House name: Animal name assigned at DLC
6	Current_Resident	Resident status: Whether or not the animal currently lives in the DLC colony.
7	StudBook	Studbook Number: Regional or global unique ID among captive individuals of that species; assigned by AZA studbook keeper. Not all individuals have studbook numbers in this data record.
8	DOB	Date of Birth: DOB is an exact date unless there is an entry in the ‘Estimated_DOB’ field.
9	Birth_Month	Month of birth: Identified from DOB
10	Estimated_DOB	Date of Birth estimate: Whether or not the date of birth is an estimate. Y=estimated to the nearest year, M to the nearest month, and D to the nearest day. If there is a number after the letter code, that indicates to the Nth value of the code. U=unknown. If there is no entry in this field, DOB is not an estimate.
11	Birth_Type	Type of birth: Whether the animal was captive-born (CB), wild-born (WB) or of unknown birth type (UNK)
12	Birth_Institution	Birth institution: Name or ISIS abbreviation of institution where animal was born. Duke Prim=DLC. For wild caught animals, birth institution=country of origin, if known.
13	Litter_Size	Litter size: Number of infants in the litter the focal animal was born into (including focal animal). Only indicated where verifiable (born at DLC). A missing value indicates that the litter size is unknown.
14	Expected_Gestation_d	Expected gestation length: Values based on DLC observations and reports from the literature, in days
15	Estimated_Concep	Date of estimated conception: Calculated as (DOB-Expected_Gestation)
16	Concep_Month	Month of estimated conception: Identified from Estimated_Concep
17	Dam_ID	Specimen ID of female parent. DLC unique ID preferred if there is one. Local ID of another ISIS-reporting institution if known and no DLC number exists. ‘Wild’ indicates dam of wild-caught individual. ‘Unk’ or no data indicates dam is unknown.
18	Dam_Name	House name of female parent (at DLC)
19	Dam_Taxon	Taxon of female parent: Based on taxonomic code described above
20	Dam_DOB	Date of birth of female parent: If female parent is wild caught or of unknown origin, this date is an estimate.
21	Dam_AgeAtConcep_y	Estimated age of female parent at conception of focal animal: in years ((Estimated_Concep-Dam_DOB)/365)
22	Sire_ID	Specimen ID of male parent: DLC number preferred if there is one. Local ID of another ISIS-reporting institution if known and no DLC number exists. ‘Wild’ indicates sire of wild-caught individual. ‘MULT’ indicates multiple possible sires. A following number indicates number of possibilities (e.g. MULT2). ‘Unk’ or no data indicates unknown sire and may include cases of multiple possible sires.
23	Sire_Name	House name of male parent (at DLC)
24	Sire_Taxon	Taxon of male parent: Based on taxonomic code described above
25	Sire_DOB	Date of birth of male parent: If male parent is wild caught or of unknown origin, this date is an estimate.
26	Sire_AgeAtConcep_y	Estimated age of male parent at conception of focal animal: in years ((Estimated_Concep-Dam_DOB)/365)
27	DOD	Date of death: Verified date an animal died. Missing indicates animal is either alive or status is unknown
28	AgeAtDeath_y	Age at death: Age of animal at verifiable date of death, in years ((DOD-DOD)/365). Missing indicates animal is either alive or status is unknown.
29	AgeOfLiving_y	Age if alive: Verifiable living age of DLC-owned animals and/or current residents at DLC on loan, in years as of the date the datafile was updated ((date of last update-DOB)/365). Missing indicates animal is either dead or status is unknown.
30	AgeLastVerified_y	Last verifiable age: Age of animal at most recent date a non-DLC owned, non-current resident animal was verifiably alive, in years. Dates were obtained from ISIS as entered by other institutions (dates of live weight or animal transfer) or via direct communication from other animal facilities. ((DateLastVerified-DOB)/365). Missing indicates animal is known to be dead or alive.
31	AgeMax_LiveOrDead_y	Maximum age: The animal's age from any of the three age categories (each individual must have a value in one of the three) indicating the maximum age the animal could have achieved as of the date the datafile was updated.
32	N_known_offspring	Number of offspring: Number of offspring the individual is known to have produced. There may be additional offspring for this individual if they were born at another institution or if this individual is a possible, rather than known, parent.

**Table 5:** DLC Weight File variable descriptions

**count**	**Weight File Variable Name**	**Weight File Variable Definition**
1	Taxon	Taxonomic code: In most cases, comprised of the first letter of the genus and the first three letters of the species; if taxonomic designation is a subspecies, comprised of the first letter of genus, species, and subspecies, and hybrids are indicated by the first three letters of the genus. See Table 1 for details.
2	Hybrid	Hybrid status: N=not a hybrid. S=species hybrid. B=subspecies hybrid. If sire is one of multiple possible and animal could be a hybrid, it is designated a hybrid.
3	DLC_ID	Specimen ID: Unique DLC number assigned at accession of animal
4	Sex	Sex: M=male. F=Female. ND=Not determined
5	Name	House name: Animal name assigned at DLC
6	DOB	Date of Birth: DOB is exact unless there is an entry in the ‘Estimated_DOB’ field.
7	Estimated_DOB	Date of Birth estimate: Whether or not the date of birth is an estimate. Y=estimated to the nearest year, M to the nearest month, and D to the nearest day. If there is a number after the letter code, that indicates to the Nth value of the code. U=unknown. If there is no data in this field, DOB is not an estimate.
8	Weight_g	Weight: Animal weight, in grams. Weights under 500 g generally to nearest 0.1-1 g; Weights >500 g generally to the nearest 1-20 g.
9	Weight_Date	Weight date: Date animal was weighed
10	MonthOfWeight	Weight month: Month of the year the animal was weighed
11	AgeAtWt_d	Age in days: Age of the animal when the weight was taken, in days (Weight_Date-DOB)
12	AgeAtWt_wk	Age in weeks: Age of the animal when the weight was taken, in weeks ((Weight_Date-DOB)/7))
13	AgeAtWt_mo	Age in months: Age of the animal when the weight was taken, in months (((Weight_Date-DOB)/365)*12)
14	AgeAtWt_mo_NoDec	Age in months with no decimal: AgeAtWt_mo value rounded down to a whole number for use in computing average individual weights (FLOOR(AgeAtWt_mo))
15	AgeAtWt_y	Age in years: Age of the animal when the weight was taken, in years ((weight date-DOB)/365)
16	Days_Since_PrevWt	Days difference: Difference, in days, between the date of this weight and the date of the animal’s previous weight
17	Change_Since_PrevWt _g	Weight difference: Difference, in grams, between this weight and the animal’s previous weight
18	Avg_Daily_WtChange_g	Average daily change: Average daily weight change, in grams, between this weight and the animal’s previous weight
19	DOD	Date of death. Verified date an animal died. Missing indicates animal is either alive or status is unknown.
20	DaysBeforeDeath	Days before death: Number of days before the animal’s death the weight was taken (DOD-Weight_Date). Missing indicates animal is either alive or status is unknown.
21	Birth_Type	Type of birth: Whether the animal was captive born (CB), wild born (WB) or of unknown birth type (UNK)
22	Birth_Institution	Birth institution: Name or ISIS abbreviation of institution where animal was born. Duke Prim=DLC. For wild caught animals, birth institution=country of origin, if known.
23	Litter_Size	Litter size: Number of infants in the litter the focal animal was born into (including focal animal). Only indicated where verifiable (born at DLC). A missing value indicates that the litter size is unknown.
24	R_Min_Dam_AgeAtConcep_y	Dam minimum age at conception, in years, for the species from the life history summary table. Used to calculate ‘Age_Category’ as described below.
25	Age_Category	Age category: IJ (infant or juvenile): (AgeAtWt_yr<R_Min_Dam_AgeAtConcep_yr). Young-adult: (Min_Dam_AgeAtConcep_yr<=AgeAtWt_yr<2xMin_Dam_AgeAtConcep_yr). Adult: AgeAtWt_yr>=2xMin_Dam_AgeAtConcep_yr
26	Current_Resident	Resident status: Whether or not the animal currently lives in the DLC colony
27	Preg_Status	Pregnancy status: Whether or not animal is pregnant on date weight was taken. P=pregnant, NP=not pregnant (all males coded NP)
28	Expected_Gestation_d	Expected gestation length: Values based on DLC observations and reports from the literature, in days
29	ConcepDate_IfPreg	Conception date: Estimated date of infant conception if the weight was taken while a female was pregnant
30	InfantDOB_IfPreg	Infant Date of Birth: Date of infant birth if the weight was taken while a female was pregnant
31	DaysBeforeInfBirth_IfPreg	Days until birth: Days remaining in the pregnancy if the weight was taken while a female was pregnant
32	Pct_PregRemain_IfPreg	% of pregnancy remaining: Percentage of pregnancy remaining when weight was taken from a pregnant female calculated as (days_BF_inf_birth/Expected_Gestation)
33	InfantLitSz_IfPreg	Infant litter size: Number of infants in the litter a female produced if she was pregnant on date weight was taken
